# Crystal Structure of the Stress-Inducible Human Heat Shock Protein 70 Substrate-Binding Domain in Complex with Peptide Substrate

**DOI:** 10.1371/journal.pone.0103518

**Published:** 2014-07-24

**Authors:** Pingfeng Zhang, Julia I-Ju Leu, Maureen E. Murphy, Donna L. George, Ronen Marmorstein

**Affiliations:** 1 Program in Gene Expression and Regulation, The Wistar Institute, Philadelphia, Pennsylvania, United States of America; 2 Department of Biochemistry & Biophysics, Abramson Family Cancer Research Institute, Perelman School of Medicine at the University of Pennsylvania, Philadelphia, Pennsylvania, United States of America; 3 Department of Genetics, Perelman School of Medicine at the University of Pennsylvania, Philadelphia, Pennsylvania, United States of America; 4 Program in Molecular and Cellular Oncogenesis, The Wistar Institute, Philadelphia, Pennsylvania, United States of America; 5 Department of Chemistry, University of Pennsylvania, Philadelphia, Pennsylvania, United States of America; Yale University School of Medicine, United States of America

## Abstract

The HSP70 family of molecular chaperones function to maintain protein quality control and homeostasis. The major stress-induced form, HSP70 (also called HSP72 or HSPA1A) is considered an important anti-cancer drug target because it is constitutively overexpressed in a number of human cancers and promotes cancer cell survival. All HSP70 family members contain two functional domains: an N-terminal nucleotide binding domain (NBD) and a C-terminal protein substrate-binding domain (SBD); the latter is subdivided into SBDα and SBDβ subdomains. The NBD and SBD structures of the bacterial ortholog, DnaK, have been characterized, but only the isolated NBD and SBDα segments of eukaryotic HSP70 proteins have been determined. Here we report the crystal structure of the substrate-bound human HSP70-SBD to 2 angstrom resolution. The overall fold of this SBD is similar to the corresponding domain in the substrate-bound DnaK structures, confirming a similar overall architecture of the orthologous bacterial and human HSP70 proteins. However, conformational differences are observed in the peptide-HSP70-SBD complex, particularly in the loop L_α, β_ that bridges SBDα to SBDβ, and the loop L_L,1_ that connects the SBD and NBD. The interaction between the SBDα and SBDβ subdomains and the mode of substrate recognition is also different between DnaK and HSP70. This suggests that differences may exist in how different HSP70 proteins recognize their respective substrates. The high-resolution structure of the substrate-bound-HSP70-SBD complex provides a molecular platform for the rational design of small molecule compounds that preferentially target this C-terminal domain, in order to modulate human HSP70 function.

## Introduction

The HSP70 family proteins represent an evolutionarily conserved group of molecular chaperones that are important for maintaining protein quality control and protein homeostasis. They were first identified more than thirty years ago in Drosophila as 70 kD proteins that were induced by heat stress or other potentially lethal stimuli [1], [2], and found to be critical for maintaining cell survival [3], [4]. Subsequently, other HSP70 family proteins were identified in both prokaryotes and eukaryotes, with many members also shown to be constitutively expressed and exhibit important housekeeping functions [5]–[8]. Among the many activities of HSP70 family proteins are the chaperoning of nascent polypeptides and unfolding of misfolded protein substrates, the facilitation of protein transport to organelles, the protection and/or dissolution of multi-protein complexes, and the targeting of some misfolded proteins for degradation [5], [6], [9]–[12]. HSP70 proteins are considered among the most conserved proteins in evolution as they are found in all kingdoms from archaebacteria to humans. While most prokaryotes have only one *HSP70* gene, some gram-negative bacteria and all eukaryotes encode several HSP70 proteins. For example, *Escherichia coli* has three HSP70 proteins: DnaK, HscA(Hsc66) and HscC(Hsc62), *Saccharomyces cerevesiae* encodes at least ten family members, and *Homo sapiens* encodes at least eight paralogs [5]–[8], [13], [14]. Some family members are thought to serve tissue-specific or organelle-limited roles; some are constitutively expressed, and still others are stress-induced. Current evidence suggests that certain HSP70 family members may serve overlapping, or specific, functions in a cell or organism [6], [15], [16]. Thus, a better understanding of the functional diversity of the HSP70 family proteins would benefit from greater insight regarding their structure-activity relationships.

The stress inducible human protein HSP70 (also called HSPA1A/A1B, HSP70-1 and HSP72) is of particular interest because it is considered a cancer-critical survival protein [17], [18]. Unlike the closely related, but constitutively expressed HSC70 (also known as HSPA8, Hsp70-8 and HSP73) protein, HSP70 is not essential for viability, as knockout mice for HSP70 are viable and fertile [19]. Additionally, unlike HSC70, HSP70 is expressed at very low levels in unstressed normal cells but is rapidly up-regulated under a variety of stress conditions. Importantly, it is constitutively overexpressed in most human cancer cells, and its elevated expression correlates with resistance to therapy and poor prognosis [6], [13], [15], [16]. Evidence indicates that, among its cancer-supporting activities, HSP70 protects cells from apoptosis and the proteotoxic stress associated with oncoproteins and aberrant metabolism [20]–[22]. Accordingly, this molecular chaperone has emerged as an attractive therapeutic target, and several groups have focused efforts on the identification of HSP70 inhibitors for use in cancer therapy. To date, however, relatively few effective, well-characterized modulators of HSP70 activities have been reported [7], [8], [22]–[30]. In general, a lack of structural information on the human HSP70 protein has slowed the development of more effective, clinically useful inhibitors. It is expected that generating new structural information on human HSP70 should aid in the development of such modulators.

HSP70 binds to small hydrophobic stretches of amino acids in nascent or partially folded substrates; together with the actions of critical co-chaperones, it directs the substrates to a particular fate, such as re-folding or degradation. HSP70 proteins share a similar overall structure, comprised of an N-terminal nucleotide binding domain (NBD) that, on its own, exhibits modest ATPase activity, and a C-terminal peptide substrate-binding domain (SBD). The actions of the two major domains of the HSP70 proteins are allosterically regulated. In the presence of ADP, model substrate peptides such as NRLLLTG or client proteins interact with high affinity. However, when ATP is bound to the NBD, substrate binds significantly more weakly. The NBD is subdivided into four subdomains, which are partitioned into two lobes (I and II) by a central ATP/ADP binding pocket [31], [32]. The SBD is composed of a two-layered β-sandwich (SBDβ), which contains the peptide binding pocket, and an α-helical subdomain (SBDα) that functions as a lid, covering the substrate binding cleft [33], [34]. The SBDβ and SBDα subdomains are connected by the loop L_α,β_, which shows limited species conservation. The NBD and SBD are bridged by a highly conserved interdomain linker (also known as loop L_L,1_), which has been implicated as being critically involved in modulating the allosteric regulation of HSP70 proteins [35]–[39]. The extreme C-terminal domain of HSP70 is believed to be largely unstructured, and is the docking site for some co-chaperones [6].

Among the HSP70 family proteins, *E. coli* DnaK has been the most extensively studied member at the structural level, though there is some structural information about the mammalian heat shock cognate (HSC70) member as well. Indeed, much of our mechanistic understanding of HSP70 structure and activity has come from analyses of *E. coli* DnaK. Several structures of the DnaK NBD [40] and SBD region, either alone or in complex with various client peptides [33], [34], [41]–[44], have been determined. Several near full-length DnaK structures, bound to either ADP or ATP in the presence or absence of substrates, were also more recently determined [37], [39], [45], [46]. In the past two decades, several structures of isolated NBDs of HSC70 in the absence of nucleotides and in the presence of ADP or ATP as well as the isolated SBDα subdomain of HSC70 have been determined [45], [47]–[51]. Finally, a near full-length, nucleotide-free bovine HSC70 X-ray structure (residues 1-54; PDB code 1YUW) has been reported [52].

Significantly less is known about the structure of the stress-inducible human HSP70, particularly of the SBD. To date, the following structures of human HSP70 have been determined: the nucleotide-bound or apo-form NBD [53]–[58], the isolated NBD in complex with the inhibitor VER-155008 (PDB code 4IO8) [59], and the isolated C-terminal SBDα (residues 537–610 [60]; or residues 534–615, PDB code 3LOF). No structure is available of a complete HSP70 SBD, which includes both SBDβ and SBDα, and no structure has been reported of the HSP70 SBD in complex with a substrate. Here we present the 2 Å crystal structure of the stress inducible human HSP70 (residues 386–616) bound to a hepta-peptide model substrate, NRLLLTG, used in previous DnaK protein structural analyses. This high-resolution structure represents the first of an intact eukaryotic HSP70 SBD; it provides a previously unavailable molecular platform to use in structure-function analyses and should aid in the rational design of HSP70-specific modulators that preferentially target the SBD of this important molecular chaperone.

## Materials and Methods

### Protein Expression and Purification

Constructs of human HSP70 (residues 1–641, 386–616, 391–615, and 395–510) were cloned into pET25 (EMD Millipore Chemicals, Inc., Billerica, MA, USA), between the *NdeI* and *XhoI* restriction sites, for expression as N-terminal His_6_-tagged fusion proteins in *E. coli*. All plasmid inserts were verified by DNA sequencing. The *E. coli* BL21 Star (DE3) competent cells (Invitrogen catalog number C6010-03) were transformed with the respective expression plasmids. The resulting strains were grown at 37°C in LB medium containing 50 µg/ml of carbenicillin (Sigma-Aldrich Co., St. Louis, MO, USA). At an OD_600_ ∼0.3–0.5, protein expression was induced with 0.5 mM IPTG, and cells were subsequently grown overnight at 25°C. Cells were collected by centrifugation and resuspended in the Bugbuster Master Mix (EMD Millipore Chemicals catalog number 71456-4), supplemented with protease inhibitors, 1 mM DTT and 20 mM imidazole. The His_6_-tagged fusion proteins were isolated on Ni^2+^-chelating resins (Ni-NTA Superflow, Qiagen catalog number 30410) by standard procedures. The purified proteins were dialyzed thoroughly against 10 mM Tris-HCl (pH 7) supplemented with 5 mM DTT at 4°C. When necessary, size-exclusion chromatography on a Superdex-200 or a Superose 6 10/300 GL (GE Healthcare) analytical column pre-equilibrated with 10 mM Tris-HCl (pH 7) and 5 mM DTT was used as a final purification step. The pure proteins were concentrated and microcentrifugated at 15,000×g for 5 min at 4°C, the concentration was determined by Bradford Protein Assay and aliquots of the soluble proteins were stored at −80°C.

### Crystallization, Data Collection, and Structure Determination

We used the hanging drop vapor diffusion technique to co-crystallize the HSP70-SBD with the NRLLLTG peptide. 5 mM of NRLLLTG (Biomatik, Wilmington, Delaware, USA) was mixed with 400 µM of HSP70 protein (residues 386–616, 391–615, or 395–510) or 400 µM of Seleno-Methionine (SeMet)-derivatized HSP70 protein. The protein mixtures were preheated to 42°C for 15 minutes and gradually cooled to room temperature. The protein preparations were subsequently microcentrifugated at 15,000×g for 5 min at room temperature, and the soluble protein complexes were used to set up crystallization screens. The best crystals were obtained using a HSP70 construct containing residues 386–616 from a crystallization solution of 0.1 M Bis-Tris (pH 5.5), 0.2 M Li_2_SO4, with 28∼30% PEG3350 in the reservoir and 22%∼25% PEG3350 in the crystallization drop. The crystals grow to full size (0.05∼0.2 mm in three dimensions) in 2∼3 days and flash-frozen directly in liquid nitrogen. Data were collected using the X29A beamline at the National Synchrotron Light Source (Brookhaven National Laboratory). One data set each for native and SeMet-derivatized HSP70-SBD/substrate crystals were collected at wavelengths of 1.075 Å and 0.9791 Å (selenium peak), respectively, and then processed with HKL2000 [61]. The data were useful to resolution limits of 2 Å and 2.5 Å, respectively. Eight selenium sites from data collected from the SeMet-derivatized crystals were identified with the HYSS program [62] using the Phenix suite [63]. These sites were input into the Phenix/Phaser program [64] and protein phases were determined using the same dataset by the single-wavelength anomalous dispersion (SAD) method. A high quality electron density map was generated and a initial model of 323 amino acid within 16 fragments were build using Phenix/Autobuild [65] automatically. The structure was refined and manually adjusted using the high resolution native dataset by iterative cycles of refinement with Phenix/Refine [66] and model building with Coot [67] (Table 1). All the structural alignments and figures were prepared with Pymol (http://www.pymol.org).

**Table 1:** Data Collection and Refinement Statistics.

	SeMet-HSP70-NRLLLTG	Native HSP70-NRLLLTG
**Data collection**		
Space group	C2	C2
Cell dimensions		
* a*, *b*, *c* (Å)	100.25, 84.80, 72.15	100.29, 85.28, 72.20
α, β, γ (°)	90.00, 127.20, 90.00	90.00, 126.97, 90.00
Wavelength	0.97910	1.0750
Resolution (Å)	50–2.50 (2.59–2.50)	50–2.0 (2.07–2.00)
R_merge_	8.9 (31.9)	9.9 (24.5)
I/σ (I)	20.8 (6.7)	20.7 (8.1)
Data completeness (%)	100.0 (100.0)	99.8 (99.4)
Redundancy	7.4 (7.5)	7.5 (7.5)
No. reflections	16817	32837
**Refinement**		
Resolution (Å)		50–2.0 (2.07–2.00)
No. reflections		32790
R_work_/R_free_		17.54/21.78
Molecules/a.u.		2
No. atoms		4105
Protein		3448
Peptide		99
Water		543
B-factor (Å^2^)		
Average		29.59
Protein		28.12
Peptide		30.89
Water		37.07
r.m.s deviations		
Bond lengths (Å)		0.0040
Bond angles (°)		0.842
Ramachandran plot (%)		
Favored regions		99.3
Additionally allowed regions		0.7

r.m.s., root-mean-square deviation. Values in parenthesis are for the highest resolution shell.

### Protein Data Bank accession code

Atomic coordinates for the human HSP70 SBD structure have been deposited with the Protein Data Bank under the identifier code 4po2.

## Results and Discussion

### Overall Structure of the Human NRLLLTG-Bound HSP70-SBD

**Figure 1:**
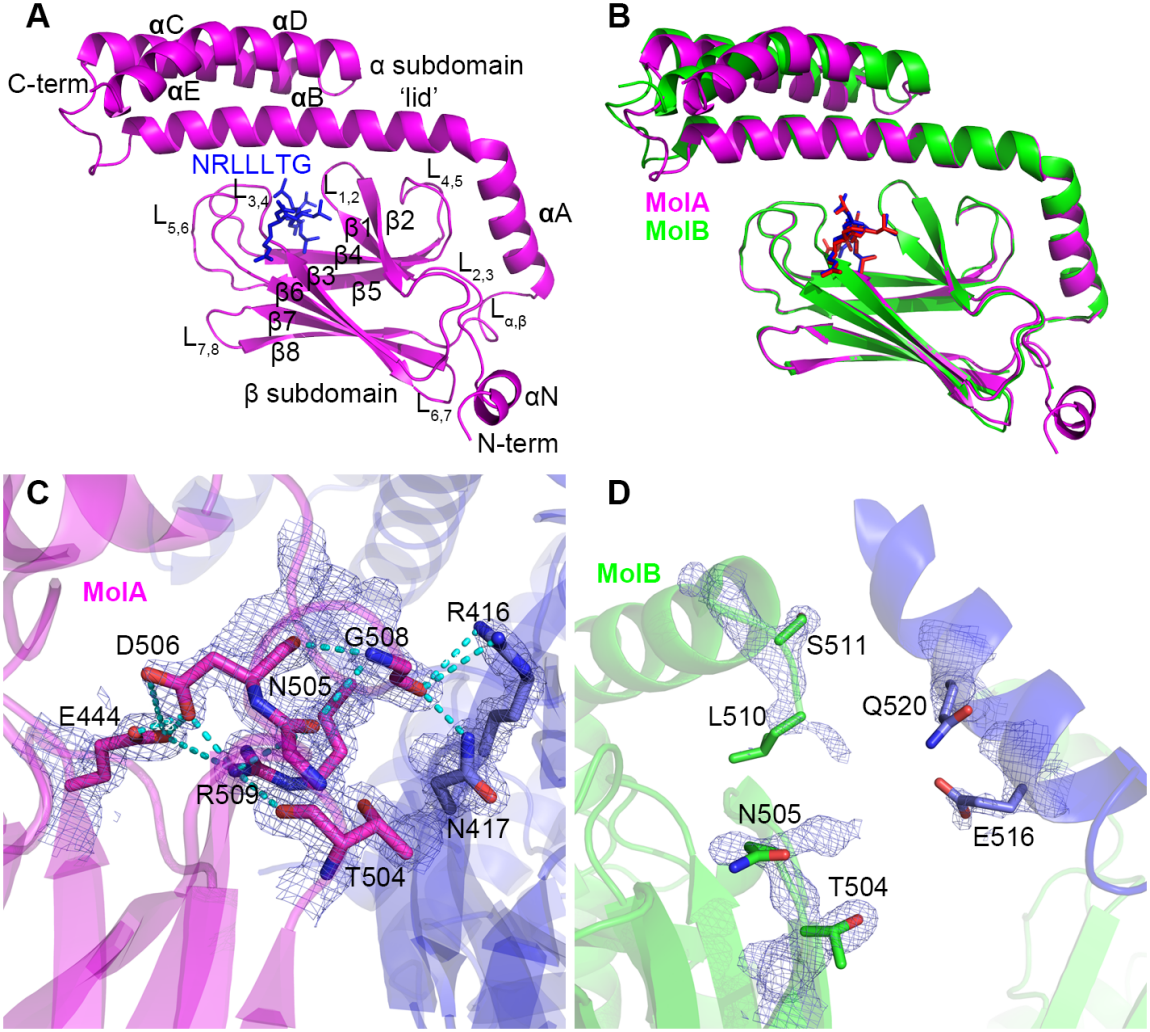
Overall structure of the human peptide-bound HSP70 SBD. A. Cartoon representation of NRLLLTG-bound HSP70 SBD Molecule A. The SBD and NRLLLTG peptide are shown in magenta and blue, respectively, and all domains and secondary structural elements are labeled. B. Superposition of molecules A and B in the asymmetric unit cell. Molecules A and B are color-coded magenta and green, respectively. C and D. Hinge region between the α and β subdomains. Molecules A and B are shown in panels C and D, respectively. Interacting (MolA) or potential interacting (MolB) residues are indicated in stick and CPK coloring (oxygen in red and nitrogen in blue). The electron density maps corresponding to these residues are contoured at 1.5 (MolA) and 1.0 (MolB) sigma. Hydrogen bonds are indicated with dotted lines.

We set out to determine the high-resolution crystal structure of the human HSP70 SBD bound to a client peptide substrate. Towards that goal, we were able to prepare well-ordered crystals of HSP70 (aa 386–616) bound to a NRLLLTG peptide that formed in the C2 space group and diffracted to 2 Å resolution. The structure of the complex was determined with single anomalous diffraction using selenomethionine-derivatized protein and refined to 2 Å to R_work_ and R_free_ values of 17.54% and 21.78%, respectively, with excellent stereochemistry (Fig. 1A and Table 1).

The X-ray crystal structure of NRLLLTG-bound HSP70-SBD reveals two molecules of the complex per asymmetric unit cell. Each molecule contains the characteristic SBDβ and SBDα subdomains (Fig. 1A), as previously observed in the NRLLLTG-bound DnaK-SBD [33]. Molecule A (MolA) can be traced from Asn387 to Gly613, while Molecule B (MolB) can only be traced from Asp395 to Gly613 and contains a gap in the electron density map corresponding to residues 506–509 in the loop L_α,β_ region. Based on sequence alignment, the human HSP70 SBD domain (residues 391–615) corresponds to residues 389–607 of DnaK, which was used previously for structural studies [33]. Since we employed residues 386–616 of human HSP70 for structure determination here, the human HSP70 SBD structure is five amino acids longer at the N-terminus than the DnaK SBD previously reported [33]. Additionally, we retained the 6-his tag at the N-terminus for crystallization. This N-terminal region of the HSP70 SBD, which represents the interdomain linker between the NBD and SBD, forms a short helix on MolA (referred to here as αN) but is disordered in MolB. These differences are likely mediated by the crystal environment since the N-terminus of MolA forms crystal contacts while the corresponding region of MolB does not. The corresponding region of intact *E.coli* DnaK forms a β strand [39], and the corresponding region of ADP-bound intact *Geobacillus kaustophilus HTA426* DnaK (gkDnaK) forms an extended loop structure [45]; it is also distinct from a loop in the nucleotide free NBD of the bovine HSC70 structure [52]. Taking this comparison together, suggests that the interdomain linker between the NBD and SBD of human HSP70 is most likely to be inherently flexible.

### Comparison of the two molecules in the asymmetrical unit cell

The overall fold of MolA and MolB in the asymmetric unit cell is essentially identical (Fig. 1B). Each monomer is composed of the SBDβ subdomain that contains the peptide-binding pocket, and an SBDα subdomain, also known as the lid. A superposition of MolA to MolB reveals that the SBDβ subdomain contains a root mean square deviation (RMSD) of 0.18 Å for 110 equivalent Cα positions. The SBDα subdomain shows more variability, particularly in the three-helix bundle of the SBDα region, with an overall RMSD of 1.7 Å for 198 equivalent Cα positions of the SBD. The latter observation is consistent with prior studies of substrate-bound DnaK structures, which indicated the existence of considerable structural variability within the SBDα region [33], [41], [42], [69].

**Figure 2:**
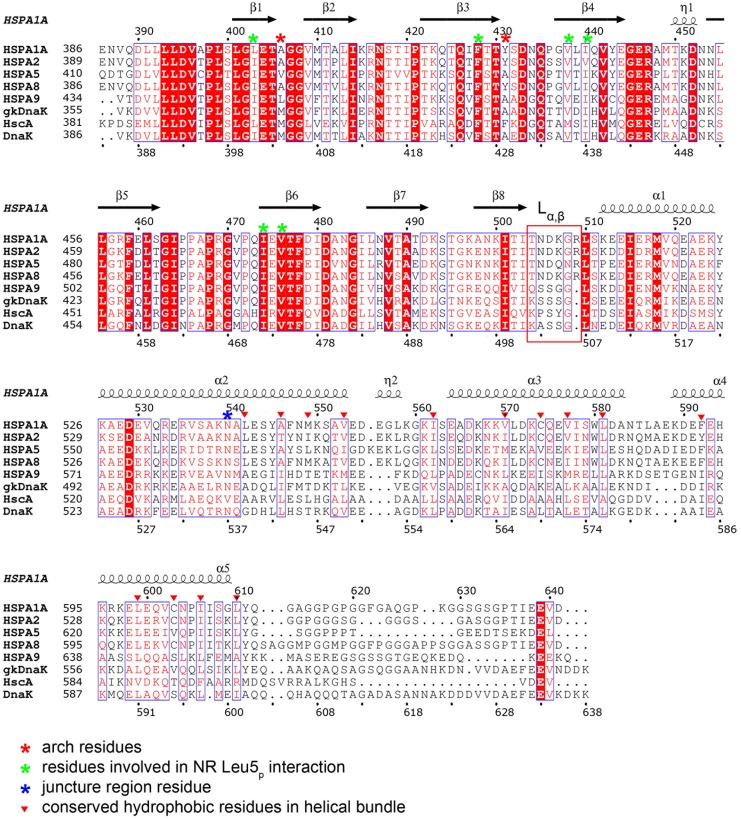
Sequence alignment of eukaryotic and bacterial HSP70 proteins. Human HSP70 (HSPA1A or HSP72), HSPA2, HSPA5 (GRP78 or BiP), HSC70 (HSPA8 or HSP73), HSPA9 (GRP75, Mortalin-2 or MTHSP70) and *E.coli* DnaK (ecDnaK), *E. coli* HscA, *Geobacillus kaustophilus HTA426* DnaK (gkDnaK) sequences are used for the alignment. Secondary structure elements and residue numbering for HSP70 is indicated above the protein sequence. The hinge region (also known as loop L_α,β_) is highlighted in a red rectangular box, the arch residues are indicated with red asterisks, residues involved in NRLLLTG Leu5_p_ substrate binding are indicated with green asterisks, and the juncture region residue Asn540 is indicated with a blue asterisk. The conserved hydrophobic residues in the helical bundle region are indicated with red triangles.

The linker region between the α and β subdomains (loop L_α,β_) is of particular interest because it has been shown in the ATP-bound DnaK structures to serve as a dynamic hinge that serves to appropriately position the long helix αB of the SBDα[37], [39]. A sequence alignment of this region reveals significant differences between *E. coli* DnaK and human HSP70s. In DnaK, this linker region is composed of residues with small side chains (ASSGL or SSSGL), while in human HSP70 this linker region is composed of conserved amino acids with larger side chains (NDKGRL) (Fig. 2). Phylogenetic analysis shows that, unlike other regions of HSP70, this region is significantly different between prokaryotes and eukaryotes [70]. In MolA of our structure, this linker is involved in crystal packing through interactions with a loop from an adjacent molecule (Fig. 1C). Specifically, the carboxylate oxygen atom of Gly508 hydrogen bonds with arginine and asparagine residues of a symmetry-related molecule. The side chain of Arg509 also folds back to interact with both the backbone of Thr504 and Asn505, and the side chain of Asp506 and Glu444. In addition, the side chain of Asp506 forms hydrogen bonds with Glu444. All these interactions hold the linker region in a fixed position, so that the electron density map in this region is very clear. In contrast, the corresponding linker region in MolB is not observed because the adjacent molecule is more than 5 Å away and, as a consequence, the electron density is poorly defined in this region of MolB (Fig. 1D). The dramatically different conformations of the linker region in the two molecules in the asymmetric unit is consistent with the inherent flexibility of this region in human HSP70.

### Comparison to other HSP70 SBD Structures

**Figure 3:**
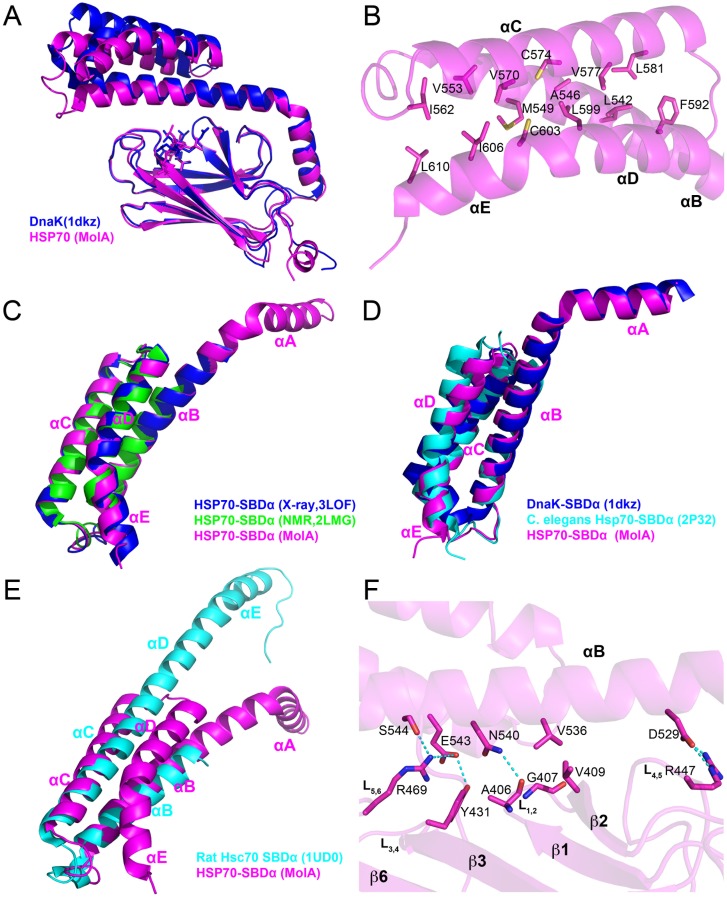
Comparison of the substrate-bound HSP70 SBD to other DnaK and HSC70 SBD structures. A. Comparison of the NRLLLTG-bound HSP70 SBD to the NRLLLTG-bound DnaK SBD (PDB code 1DKZ). HSP70 and DnaK are shown in magenta and blue cartoons, respectively. The corresponding peptide substrates are shown as stick models. B. A close-up view of the hydrophobic interactions within the helical bundle region of the α subdomain. Hydrophobic residues are shown as stick models. C. Comparison of the SBDα subdomain of the human HSP70-NRLLLTG structure with the isolated three-helix bundle region of the same protein in X-ray structure (PDB code 3LOF, blue) and NMR solution structure (PDB code 2LMG, green). D, Structural alignment of the SBDα subdomain of the HSP70-NRLLLTG structure with the three-helix bundle region of *C. elegans* Hsp70 (PDB code 2P32, cyan) and DnaK (PDB code 1DKZ, blue). E. Superposition of the lid subdomains of human HSP70 and rat HSC70. HSP70 (MolA) and HSC70 are colored in magenta and cyan, respectively. F. A close-up view of the interaction between the SBDα and SBDβ subdomains of HSP70. Interacting residues are highlighted as sticks, and hydrogen bonds are indicated with dotted lines.

The human HSP70 SBD is highly homologous to the DnaK SBD (51% sequence identity in the full-length protein and 47% identity in the SBD) (Fig. 2). Not surprisingly, the overall fold of the HSP70-NRLLLTG structure closely approximates the other substrate-bound DnaK SBD structures, with the SBDβ subdomain showing the greatest degree of structural homology with an RMSD of about 0.4 Å, depending on which DnaK structure is used for the comparison (Fig. 3A). In contrast, loop L_α,β_ is significantly different as there is one more residue inserted in this region of the human HSP70 proteins, and, as stated previously, the side chains are longer than those in DnaK. The SBDα subdomains of the peptide-bound HSP70 and DnaK structures show greater structural variability with an average RMSD of about 1.5 Å (Fig. 3A). Importantly, the modest degree of structural variation between the SBD of HSP70 and DnaK is on par with the structural variation between the two HSP70 molecules in the asymmetric unit, which is consistent with the highly conserved structures of the SBD of both human HSP70 and bacterial DnaK. This observation is in contrast to previous observations based on partial HSC70 SBD structures, which suggested that the HSC70 SBDα region harbors considerable differences to the corresponding region of DnaK [49], [52].

The SBDα subdomain is comprised of five α-helices (αA–αE). The helix αA connects the loop L_α,β_ to the αB helix, the C-terminal half of which forms an anti-parallel three-helix bundle with helices αC and αD. The αD helix is contiguous with the αE helix. The structure of HSP70 reveals that the helices within the lid are stabilized by extensive van der Waals interactions between hydrophobic residues (Fig. 3B). These hydrophobic residues are not strictly conserved (including Leu542, Ala546, Met549, Val553, Ile562, Val570, Cys574, Val577, Leu581, Phe592, Leu599, Cys603, Ile606, Leu610 in HSP70), but the corresponding positions in DnaK are always occupied by hydrophobic residues (Figs. 2). The helix αE in the HSP70-NRLLLTG structure is kinked by about 30° because of the hydrophobic interaction between this helix and the three helical bundle. This is significantly different from helix αE of crystalized DnaK (which is 1 residue shorter at the C-terminus than the human HSP70 SBD structure reported here), which is kinked by about 60°. Specifically, our findings are most consistent with the following eukaryotic HSP70 SBDα structures: the crystal structures of the human HSP70-SBDα helical bundle (residues 534–613; PDB code 3LOF; overall Cα RMSD = 0.42 Å); the solution structure of human HSP70 SBDα helical bundle (residues 537–610; PDB code 2LMG; overall Cα RMSD = 0.77Å) [60] (Fig. 3C); and the *C. elegans* Hsp70 SBDα helical bundle (residues 533–614; PDB code 2P32; overall Cα RMSD = 0.58 Å) [71] (Fig. 3D). Based on the comparison of these structures, it appears that eukaryotic HSP70 proteins have a different helix αE conformation than DnaK.

The previous crystal structure of the rat HSC70 lid region revealed a dramatically different organization of the helices, in which helices αC, αD and αE are combined to form a long helix [49] (Fig. 3E). Although this structure suggested that the SBDα of HSC70 is structurally distinct from that of DnaK, our structure shows that human HSP70 adopts the same SBDα fold as DnaK. The different helix organization in HSC70 might be influenced by the truncated construct used or the crystal lattice environment. This conclusion is bolstered by the analogous structures of the three isolated three-helix bundle structures from eukaryotic HSP70 discussed above. Taken together, this comparison is consistent with a structurally conserved and rigid lid region between the eukaryotic and bacterial HSP70 proteins.

The combined structural data support the notion that the SBDα subdomain is inherently more flexible than the SBDβ [33], [42], [69]. This is even true when one compares the SBDα subdomain from different molecules in the asymmetric unit of the same crystal structure, as illustrated in the comparison between MolA and MolB of HSP70 reported here (Fig. 1B). On closer inspection, it appears that the junction for this variability occurs around the center of helix αB where this helix participates in the helical bundle of the SBDα subdomain (Fig. 3F). In particular, consistent with the DnaK juncture region (536–538) [33], the juncture point for this region appears to be around residue Asn540. This residue is identical in most HSP70 proteins (except *E. coli* HscA) and also makes conserved interactions with residue Ala406 on the SBDβ subdomain. In this HSP70 structure, Asn540 interacts with the backbone of Ala406 (3.3 Å in MolA, 3.0 Å in MolB), which is in the L_1,2_ loop forming the substrate-binding groove on the SBDβ subdomain. A helical turn away, Val536, which is either a valine or isoleucine residue in HSP70 proteins, makes van der Waals contacts with Gly407 and Val409; notably, Gly407 is strictly conserved in all HSP70s and Val409 is conserved on strand β2 of the SBDβ subdomain in most HSP70 proteins, except for *E. coli* HscA (Fig. 2). Val536 is always placed in between Gly407 and Val409. Additional hydrogen bond interactions occur between Asp529 at the N-terminal end of helix αB and Arg447 of loop L_4,5_ of the SBDβ subdomain. Together, these residues are highly conserved in HSP70 proteins. These conserved interactions fix the conformation of the N-terminal half of helix αB. However, the interactions in the C-terminal part of helix αB are less conserved. For instance, in MolA, residues Glu543 and Ser544, one helical turn C-terminal to Asn540 of helix αB, hydrogen bond with Arg469 of loop L_5,6_ and Tyr431 of loop L_3,4_ of the SBDβ subdomain (Fig. 3F). In contrast, these interactions differ in MolB, with Arg469 adopting a different conformation that cannot contact Glu543 and Ser544. Since Arg469 extends to Phe547, it appears that MolB is forming a destabilizing interaction in this region. Together, this network of interactions between the N-terminal end of helix αB of the SBDα subdomain and the loops of the SBDβ subdomain serve to rigidly link this region of helix αB as well as the preceding helix αA to the SBDβ subdomain, but leaves the C-terminal part of helix αB and the associated helical bundle region of the SBDα subdomain to take on more divergent positions when compared to different HSP70 proteins. Interestingly, DnaK shows a more extensive network of interactions between residues within helix αB and the SBDβ subdomain. This is consistent with the greater degree of structural and functional plasticity of the eukaryotic HSP70 proteins relative to the DnaK proteins. We hypothesize that the less constrained SBDα subdomain of the HSP70 proteins may influence the interaction of HSP70 proteins with different co-chaperones such as CHIP [72].

### Substrate Recognition by HSP70

**Figure 4:**
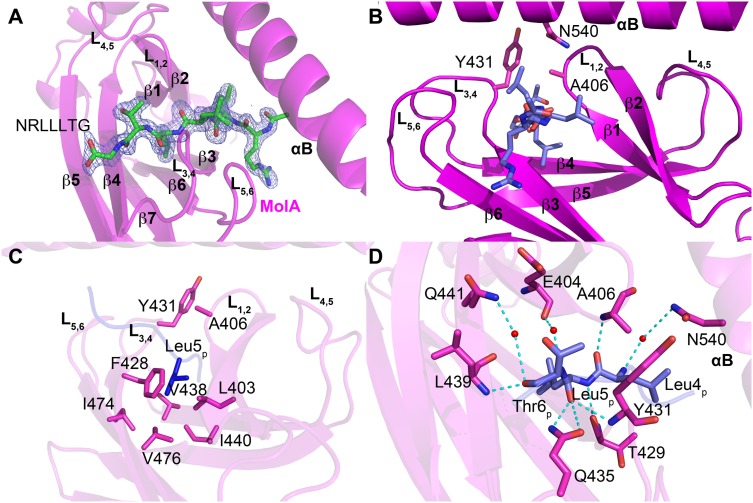
NRLLLTG peptide substrate binding by HSP70. A. Omit electron density of the peptide substrate bound to the β subdomain of molecule A is contoured at 3.0 sigma. The NRLLLTG peptide is shown as a stick model in CPK coloring. B. Peptide binding site highlighting the flanking L_1,2_ and L_3,4,_ and supporting L_5,6_ and L_4,5_ loops. The NRLLLTG peptide and arch residues are shown as stick models. C. Detailed view of the NRLLLTG-binding pocket in HSP70-NRLLLTG highlighting a network of van der Waals contacts with Leu5_p_ of the NRLLLTG peptide. D. A close-up view of the interactions between Leu4_p_-Leu5_p_-Thr6_p_ of the NRLLLTG peptide and the surrounding residues in HSP70-NRLLLTG. All interacting residues are shown as sticks and hydrogen bonds are shown as dotted lines.

The NRLLLTG substrate peptide is known to bind to the ADP-bound HSP70/DnaK with high affinity [33], [73]. However, upon exchange of ADP with ATP, the HSP70/DnaK protein undergoes a conformational change, switching to the lower-affinity peptide binding state [38], [73], [74]. Similar to the NRLLLTG-DnaK-SBD structure, the NRLLLTG peptide substrate binds to the SBDβ subdomain of the human HSP70 SBD (Fig. 4A). This indicates that this structure is representative of the peptide bound to the ADP form of full length HSP70. The SBDβ subdomain is made up of two layers of β sheets that interact through hydrophobic interactions (Figs. 1A and 4A). The first layer consists of strands β3, β6, β7 and β8, and the second layer contains strands β5, β4 and β1, β2 (Fig. 1B). These β-strands are connected by loops L_1,2_ through L_6,7_. The NRLLLTG peptide binding pocket is situated between loops L_3,4_ and L_1,2_, and the binding cleft is further stabilized by loops L_4,5_ and L_5,6_ through a series of hydrogen bonds and multiple van der Waals contacts (Figs. 1A, 4A and 4B).

The substrate-binding groove of HSP70 shows significant conservation with the corresponding groove of DnaK (and other HSP70 proteins). Highly conserved residues Leu403 (Ile401 in DnaK), Phe428, Val438, Ile440, Ile474 and Val476 at the base of the hydrophobic pocket each make van der Waals interactions with the central hydrophobic residue of the peptide substrate, Leu5_p_ (Fig. 4C). These interactions explain the strong preference for a hydrophobic residue, and particularly leucine, in this position of HSP70 substrates. The flanking peptide residues make more minor interactions with HSP70, while the side chains of Ala406 and Tyr431 form a structurally conserved arch over the surface of the pocket and make van der Waals interactions with flanking residues Leu4_p_ and Thr6_p_ (methyl group) (Fig. 4D). Positions corresponding to Ala406 and Tyr431 are not conserved within DnaK but are typically hydrophobic in DnaK and other HSP70 proteins (Fig. 2). These arch residues correspond to residues Met404 and Ala429 in *E. coli* DnaK, Met and Phe in *E. coli* HscA and Ser and Ala in *E. coli* HscC. Taken together, the HSP70 substrate-binding site appears to be tailored for a hydrophobic residue and leucine in particular that is flanked by 2 additional residues with hydrophobic character, and this is conserved in DnaK and other HSP70 proteins.

A survey of DnaK SBD X-ray structures with the same peptide sequence (i.e. NRLLLTG) reveals that most substrate-bound structures contain Leu4_p_ in the hydrophobic pocket [33], [42]. However, one structure (PDB code 4EZW) also has Leu5_p_ modeled into the pocket; and another structure contains the peptide NRLILTG instead of the canonical NRLLLTG peptide, but bound in the reverse orientation with Leu3_p_ in the hydrophobic pocket (PDB code 4EZY) [42]. Analysis of the crystal packing arrangements of several DnaK SBD/peptide structures does reveal that the crystal-packing contacts could, and likely do, influence the register of peptide binding [33], [42]. Consistent with their broad substrate specificity, we hypothesize that the HSP70 isoforms can accommodate various peptide registers, and the actual binding mode may depend on the composition of the substrate and the microenvironment.

### Implications for Developing HSP70 Inhibitors

Rodina et al. (2013) recently unveiled 5 potential druggable clefts from a theoretical model of full length human HSP70, which was generated from the DnaK SBD. Combining the S-score, D-score and size of the cavity, they identified 2 sites (sites 1 and 2) in the NBD that are considered “most druggable” (Rodina et al., 2013). Furthermore, they tested a novel inhibitor called YK5 and provided evidence, in addition to computer modeling, that it binds to site 1 [30]. Intriguingly, Schlecht et al. recently cocrystallized the N-terminal NBD domain of HSP70 (residues 1–382) with the HSP70 inhibitor VER-155008 (PDB code 4IO8) [59], and in this structure the inhibitor binds to site 2 in the NBD.

**Figure 5:**
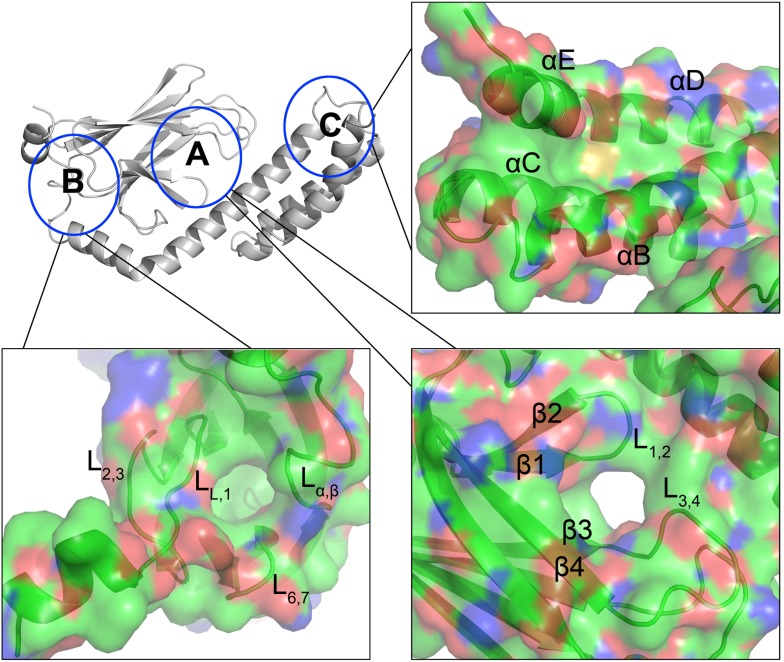
Putative druggable sites in the HSP70 SBD domain. An overview of three hydrophobic pockets within the SBD domain have been circled and marked as A, B and C. Close-up of the surface area of these three sites are illustrated in CPK coloring mode, with carbon in green, oxygen in red and nitrogen in blue. Secondary structural elements within the area are labeled. The NRLLLTG substrate is removed from this figure (pocket A) to better illustrate the pocket A for comparison with the other pockets (B and C).

The high resolution NRLLLTG-bound HSP70-SBD structure reported here provides an opportunity to identify additional potential druggable sites within the HSP70 SBD domain. A detailed analysis of the structure reveals three potentially druggable hydrophobic pockets for the rational development of inhibitors (Fig. 5), two of which were previously noted using the theoretical model of the human SBD (Rodina et al., 2013). The first site (labeled A in Fig. 5) maps to the canonical substrate-binding site, referred to here as the Leu5_p_ binding pocket. The second site (labeled B in Fig. 5) is distinct from the NRLLLTG-binding cleft and is supported by loops L_α,β_, L_2,3_, L_6,7_ and L_L,1_; a similar hydrophobic binding site has been reported by Cellitti et al. (2009) [68]. A third site (labeled C in Fig. 5) is located in the helix bundle region, which is more covert and has been investigated recently [75]. Given the importance of these predicted hydrophobic pockets in orchestrating the crosstalk among the NBD, SBDβ and SBDα domains, and the interaction between the SBD domain and other co-chaperones, it is anticipated that small molecule inhibitors or inhibitory peptides that preferentially interact with one or more of these sites might negatively impact HSP70-clients interactions.

In summary, we report the crystal structure of the complete stress-inducible human HSP70-SBD in complex with a peptide substrate at atomic resolution. This structure provides the first substrate-bound HSP70-SBD molecular scaffold for a structure-activity based strategy to understand HSP70-substrate interactions and subsequent signaling events. Analysis of the NRLLLTG-bound HSP70-SBD structure also provides intriguing clues for potential drug discovery and development.
